# Shared understandings of vaccine hesitancy: How perceived risk and trust in vaccination frame individuals’ vaccine acceptance

**DOI:** 10.1371/journal.pone.0276519

**Published:** 2022-10-21

**Authors:** Mauro Martinelli, Giuseppe Alessandro Veltri

**Affiliations:** Department of Sociology and Social Research, University of Trento, Trento, Italy; Roma Tre University: Universita degli Studi Roma Tre, ITALY

## Abstract

Extensive research has framed vaccine hesitancy as a property of a heterogeneous group of individuals, ranging from total acceptance to complete refusal. Nevertheless, not much research has explored this heterogeneity, mainly focusing on central tendencies of single belief-related items. Using data from an original survey on a sample of Italian citizens, this paper examines this heterogeneity, exploiting individuals’ cognitive variation to map clusters of individuals who share similar cognitive schemas on vaccine uptake. The results showed the existence three groups, characterized by a different articulation of predictors of vaccine hesitancy, revealing different understandings of vaccine uptake. We then analyzed within-cluster characteristics and showed that cognitive segmentation was connected to different levels of perceived risk, confidence, and support for vaccination. We further showed that cognitive clustering also entailed a mean of social stratification that was correlated with individuals’ educational levels, and that the predictors of vaccine hesitancy were articulated differently in each group. This study, adopting a recent perspective in the analysis of systems of beliefs, moves one step further in disentangling the complexity of vaccine acceptance. Results suggested the usefulness of including individuals’ cognitive characteristics in vaccine hesitancy research and in the development of interventions addressed at increasing vaccine acceptance.

## Introduction

Vaccine prophylaxis is a major public health success of the 20th century, preventing around 2 to 3 million deaths every year [1]. Although it can count on strong public support, it is not a process without challenges. Vaccine hesitancy, the delay or refusal of vaccine prophylaxis [2], has re-emerged as an issue, especially in places that more than others have seen vaccination’s beneficial effects [3]. Skepticism toward vaccines is not a recent phenomenon, but a contemporary resurgence of the issue seems to be particularly severe, enough to be included in the 2019 13th WHO General Programmes of Work, right next to Ebola and the climate crisis [4]. This phenomenon is even more critical in the light of the COVID-19 pandemic, since vaccine acceptance is fundamental to limit the spread of the SARS-CoV-2 virus. Unfortunately, recent research suggests that distrust in vaccine safety and efficacy is still a major issue [5, 6].

Vaccine acceptance is often imagined as a homogeneous debate between two oppositional factions, pro versus against, acceptance versus refusal. However, research has shown that vaccine hesitancy may be based on very different reasons, attitudes, and ideas. The recognition of this complexity also calls for greater efforts to disentangle it. Social psychology and cognitive sociology suggest that groups of individuals can be characterized by implicit epistemic frameworks of the same social object. In other words, not everyone organize their thinking about the world in the same way [7], so it is possible that groups of individuals frame their understanding of vaccination in consistently different fashions. Prior empirical research might have overlooked this complexity and the different lenses people used to interpret this issue. To address this gap, following recent works from Goldberg [7], Baldassarri and Goldberg [8], Boutyline [9], and DiMaggio et al. [10], we investigated whether different epistemologies regarding vaccine acceptance characterize groups of individuals, and we further explored if this segmentation also entailed a mean of social stratification and a differential propensity to accept vaccinations.

We considered this issue in the case of Italy, a high-income country with significant levels of vaccine skepticism, using data from an original survey implemented between September and November 2019.

This paper contributes to our understanding of vaccine hesitancy by investigating whether individuals’ heterogeneity can be linked not only to single preferences but also to wider epistemologies and worldviews. This would advocate for the relevance of analyzing people’s ideas as relational systems and the importance of exploring whether cognitive segmentation might be a mean of social distinction. At the same time, understanding vaccine hesitancy is a fundamental step to developing better strategies to increase vaccine acceptance. Paying more attention to how people develop certain worldviews, and how these are structured, can support the development of more systematic ways to help people understand the benefits of immunization.

### Socio-demographic determinants of vaccine hesitancy

Vaccine acceptance is a complex issue at the intersection of individual decisions and societal needs. Reasons behind vaccine hesitancy are still being explored, but researchers have identified an extensive set of elements empirically proven to affect vaccine uptake, including contextual, organizational and individual ones [11]. The investigation of the association between individual socio-demographic characteristics and vaccine acceptance is a central focus of vaccine hesitancy research [12]. In recent studies, scholars focused on several predictors, with special attention to individuals’ socio-economic status and educational level, but with inconsistent results [13]. Several studies showed a positive correlation between SES and immunization status [14], whereas others showed a negative one [15]. Several studies found a negative association between educational level and vaccine hesitancy [16, 17], while others found the opposite [18, 19] or that vaccine hesitancy is higher at extremes of the spectrum of both education and income [20]. Larger-scale studies suggest that this relationship might be strongly context-dependent, at both national and local levels [12]. In this complex empirical scenario, studies of the Italian context are extremely limited and inconclusive [13, 19].

At the same time, a growing trend suggests that, whereas in the past vaccines were mostly refused by lower educated people [11], today’s hesitant individuals might also be well-educated people claiming the right to make an “informed” decision about vaccination [21]. Experience-based health information has gained a level of legitimacy and credibility similar to evidence-based scientific information. Dubé et al. [11] highlighted a growing trend to seek information on user-generated websites rather than traditional evidence-based information sources. Online narratives are interesting, memorable, and in-demand [22], whereas information from official sources tend to be more cryptic and easier to forget [23].

This paradigmatic change calls into play how individuals select, retrieve, and use information to develop beliefs and attitudes on vaccine-related issues. Recent literature suggests that beyond socio-demographic characteristics, the cornerstone issue to be investigated might be the very process by which people develop their worldviews by deeming different information sources as trustworthy [24]. Given that there exists no method or form of evidence guaranteeing the way we judge a certain evidence as convincing [24], the problem might move from what people believe to how people construct meaning and share a similar epistemology with other individuals.

### Conceptualizing and measuring cognitive schemas

The key to this intricate problem might come from recent developments in cognitive science and from the coordinated study of culture and cognition. Studies of the brain have determined that external and internal inputs generate activation patterns across neurons, revealing the brain’s representational and processing mechanisms. The reification of activation patterns leads to the formation of cognitive schemas, abstract cognitive structures acquired through experience and exposure to cultural contents [9]. Cognitive schemas embody our taken-for-granted assumptions about the world under conditions of incomplete information [25]. They allow to easily represent people’s, places’, objects’ or events’ characteristics, and to deduce how these entities act and what to expect of them [26]. In other words, cognitive schemas represent contextually and culturally defined “lenses” through which individuals interpret the reality surrounding them [27]. These schemas are in turn used to organize, process, and retrieve experiences [28]. Cognitive schemas are strongly context-dependent, and for this reason, they might be both a part of an individual and distributed among groups of individuals. Individuals sharing the same cultural milieu are more likely to share similar cognitive schemas, belonging to what Fleck [29] called “thought communities”: groups of individuals who share the same worldview on a specific social object. Berger and Luckmann [30] argued that culture does not exist as an abstract entity entirely external to individuals. Rather, it is both individuated and socially distributed. Merton’s [31] notion of “pluralistic ignorance” suggests that people act on the basis of a shared representation of collective opinions. If belonging to a particular thought community implies that its members mediate their experiences using similar cognitive building blocks, then it is reasonable to assume they also employ similar reasoning patterns in understanding and responding to the realities they encounter [7]. Finally, cognitive schemas are relational systems, where the elements are linked one another through some form of functional interdependence [32]. Theories referring to relational networks suggest that the meaning of symbols in a cultural system rests not in the signs themselves, but in the relationships between them.

Applying these concepts to vaccine uptake, the heterogeneity of vaccine-hesitant individuals might be seen as comprising multiple groups of individuals whose mental representation of the issue is structured similarly, based on the pattern of relationships in each cognitive schema. If it is possible to account for this heterogeneity by showing that it is systematic and that it can be minimized within groups and maximized between clusters of individuals, this would point toward the existence of multiple belief systems on the issue of vaccine hesitancy.

### Core determinants of vaccine acceptance

Cognitive schemas are characterized by how different elements of a certain social domain are bounded together. Therefore, to understand if groups of individuals show systematically different shared understandings of vaccine acceptance, core predictors of vaccine acceptance could be used. Research has shown that the way individuals perceive the risk of vaccine preventable diseases (VPDs) and trust vaccinations are principal components in determining vaccine uptake or refusal [19, 23, 33]. These elements stand at the center of several health-specific behavioral theories which are often used to frame vaccine hesitancy (see, for example, Becker [34] and Rogers [35]).

Nearly all theories focus on two dimensions of risk perception: the perceived severity of disease and the perceived likelihood of contagion. The severity of a disease represents the magnitude of an adverse event such as VPDs [34]. The likelihood of contagion is instead the probability of being harmed by a hazard under certain behavior conditions [36]. Psychometric tradition and cultural sociology recently pointed out that people have a more complex conception of risk that calls into play an individual’s cognitive systems [37]. Kahan [38] suggested that on the issue of vaccine hesitancy, emotional assessments toward vaccines may trump the calculus of objective risks and benefits. That is, people base their judgments on an activity or technology—such as vaccines—not only on what they think about it but also on the way they feel about it. In this interpretation, risk is an affective state, distinct from cognitive judgment. For this reason, two additional dimensions should be considered. The first is the perceived susceptibility to disease, emphasizing an individual’s perceived vulnerability to VPDs. The second is the perceived feeling at risk, highlighting the emotional component of risk perception [39].

From an empirical perspective, perceived risk is often associated with health-protective behaviors and vaccine acceptance [3]. The centrality of this concept, declined in its different components, has been underlined even during pandemic crises, such as the 2009 H1N1 swine flu pandemic [40] or Ebola [41, 42]. The COVID-19 pandemic does not constitute an exception, and many empirical contributions recently underlined the importance of risk perceptions and their association with vaccine acceptance [43–46].

The second fundamental component of vaccine acceptance is trust in a valid coping mechanism. In vaccine hesitancy research, this is often interpreted in terms of confidence [2, 47, 48]. Confidence is a faceted construct, usually positively associated with an increase in vaccine uptake, although there have been mixed results in the literature, mainly because of conflicting definitions of the concept. There is, in fact, little consensus about the components of confidence, and several scales have been proposed to measure it [49, 50]. Following the systematization by Larson et al. [47], we define confidence as trust in the vaccine (the product), trust in the vaccinator and other health professional (the provider), and trust in those who make the decisions about vaccine provision (the policymaker).

Empirically, confidence has been multiple times associated with vaccination intentions, although showing noteworthy contextual variability [3, 51]. As for perceived risk, the importance of the relationship between confidence and vaccine acceptance has been underlined also in pandemic scenarios such as the H1N1 pandemic [52, 53], the 2018 Ebola outbreak [41] and the ongoing COVID-19 pandemic [54–56].

In this paper, we aim at moving the focus of attention from what people believe to how beliefs are organized, using predictors of vaccine acceptance to uncover whether individuals differ qualitatively in the way they construct their representation of vaccine uptake. Second, we investigate whether group segmentation also entails a means of social distinction or stratification by analyzing if socio-demographic characteristics are differently associated with the probability of belonging to each group. Finally, we analyze each group separately, to understand if different shared understandings of the issue are correlated with differences in the individuals’ propensity to be vaccine-hesitant.

Therefore, three research questions drive this paper:

Q1) Relying on measures of perceived risk and confidence, do groups of individuals show different shared cognitive schemas on vaccine acceptance?
Q2) Does the group partitioning of Q1 also represent a source of social distinction or stratification?
Q3) Is there a different association between core predictors of vaccine hesitancy and the willingness to vaccinate in each group?

## Materials and methods

### Data

Given the limited pre-pandemic availability of data on the Italian context and the specific aim of this paper, between September and November 2019 an original online survey was administered to 1008 Italian citizens between 20 and 64 years old who participated in an online panel of a major Italian survey company. The survey was approved by the Institutional Review Board of the Department of Sociology and Social Research at the University of Trento. Given that no experimental manipulation happened during the survey, the fully anonymized nature of responses provided by the survey company and the use of data only in aggregated form, no formal ethics committee approval was requested by the IRB. Panelists were sent an email containing a unique link to the survey. Before the beginning of the survey, participants were provided with a digital opt-in consent form that emphasized the study’s objectives, that their contribution was completely voluntary, provided contact information of researchers responsible for the survey, and that data was fully anonymized and treated only in aggregated form. Respondents were informed that by choosing to proceed they were giving their informed consent to participate.

We used a non-probabilistic quota sampling method, stratifying participants by gender, age, geographical location, and educational level. The number of individuals in gender, age, class, and geographical location quotas was proportional to the 2018 Italian population surveyed by the Italian National Institute of Statistics (ISTAT). Educational levels (low  =  less than 9 years of education, medium  =  between 9 and 13 years of education, and high  =  over 13 years of education) were equally distributed among respondents. To account for this sampling characteristic, probability weights were applied in the analysis. Quota sampling, although far from an ideal probabilistic sampling method, was chosen with careful consideration for the availability of research funds and research goals, primarily to map individuals’ cognitive structures. For this reason, estimates in the following analysis must be considered carefully, always taking this liability into account.

### Variables

In this section we describe the main variables used in the analysis. A complete description of survey questions, variables, and coding can be found in S1 Table. Our main predictor variables measured the perceived risk of VPDs and confidence in vaccination. We measured four distinct risk perception concepts: severity of VPDs, the likelihood of contagion, susceptibility to VPDs, and feeling at risk. Questions about perceived risk were preceded by asking respondents to imagine they would have to take care of a child, today, in Italy. In addition, the perceived likelihood of contagion and feeling at risk were preceded by asking respondents to imagine two hypothetical scenarios: whether the child received or did not receive all the Italian mandatory and recommended vaccinations. We called these conditioned questions with and without vaccination. Additional questions investigated the perceived probability and severity of side effects, the anticipated regret for the possible side effects of vaccination, or the development of VPDs following the decision not to vaccinate. Finally, confidence was measured by a question articulated in a battery of 6 statements, isolated from a comprehensive matrix of determinants of vaccine hesitancy developed by the Strategic Advisory Group on Experts working group (SAGE) on vaccine hesitancy [47]. The question investigated trust in the safety and effectiveness of vaccines (the product), trust in physicians, the scientific community, and the production chain (the providers), and trust in official authorities (the policymakers). The 19 variables used in the analysis can be partitioned into three issue domains: risk perception without vaccination, risk perception with vaccination, and confidence. We used a 7-point Likert scale as the answering option, recoding the limited number of “Don’t know” answers with the intermediate value (4). Previous research [39, 57] showed that, when assessing perceived risk, a 7-point answering scale is a more consistent predictor of vaccination intentions and behavior when compared to other answering options, and that results are consistent across genders. To maintain consistency throughout the questionnaire and to avoid increasing respondents’ cognitive load, we extended the same answering option to confidence questions.

As a measure of vaccine hesitancy, we asked respondents to rate, on a scale from 0 to 10, whether they would hesitate to administer to the hypothetical child all the Italian mandatory and recommended vaccinations. Given the skewed distribution (*M*: 3.05; *SD*: 3.5; *Mdn*: 1), the variable was dichotomized, where 0 indicated no hesitancy and 1 and above indicated hesitancy.

Socio-demographic variables surveyed the respondents’ educational level, gender, age, and whether respondents had children (childless, one child, more than one child), whether respondents had a religious affiliation (dichotomic, yes/no), their geographic area of residence (north-east, north-west, center, and south and islands) and rural or urban area of residence (metropolitan area, city/urban center, rural area).

### Methods

Accounting for heterogeneity in individuals’ cognitive schemas necessitates to examine the relationships between variables and individuals simultaneously [7]. This requires a method that compares individuals by detecting groups that vary with respect to patterns of relationship between attitudinal variables, without making assumptions on underlying individual characteristics—such as socio-demographic variables. To address this problem, we used Goldberg’s relational class analysis (RCA) [7]. RCA is a graph partitioning method based on the assumption that individuals are related to one another to the extent to which they construct meaning in a similar way. RCA measures the degree to which each pair of respondents employed the same cognitive schema, and uses this measure to create groups [9]. As a measure of schematic similarity, Goldberg [7] proposed a metric he called relationality, which measures whether the components of two vectors of the same set of variables follow a similar pattern. Intuitively, two individuals are as schematically more alike as their response pattern is closer. Boutyline [9] ultimately shows that relationality measures the degree of linear dependency between two individuals’ vectors of responses. RCA computes relationality for each pair of items, for each pair of observations in the dataset, resulting in a complete, undirected, weighted graph. The vertices of the graph represent individual respondents, and edge weights the average relationality between all couple of respondents in the dataset. Finally, individuals are divided into groups of schematically similar respondents using a partition maximization algorithm. Belonging to the same group does not imply having identical opinions, though. Two individuals can have very different opinions but agree that the issue is structured in a similar way. For example, a democratic and a republican will probably have very different ideas, but they might agree that the debate arena is structured in a particular way. There is a continuum with two extremes, the democratic and republican parties, inside a democratic framework. For this reason, groups individuated by RCA may contain individuals with different attitudes, but that recognize a similar relation of closeness or opposition between the items making up a specific issue domain. Each group is therefore characterized by a distinctive pattern of relationship between opinions, suggesting that group members organize their understanding of a certain social object in a similar way [8]. A more detailed explanation of RCA is available in S1 File. For a complete description of the characteristics and functioning of the RCA, see Goldberg [7].

In the second part of the analysis, we used multivariate multinomial logistic regression to model the probability of belonging to each group as a function of an individual’s socio-demographic characteristics. The aim was to understand if group segmentation also entailed a means of social stratification.

Finally, in the third part, we used multivariate logistic regression to establish whether individual characteristics and perceptions correlated differently with vaccine acceptance in each group.

The first part of the analysis relied on the software R Studio and the package ‘RCA’ (2.0), while the following analyses have been carried out using the software Stata, version 16.1.

## Results

### Relational class analysis: Convinced, Skeptics, and Agnostics

**Fig 1:**
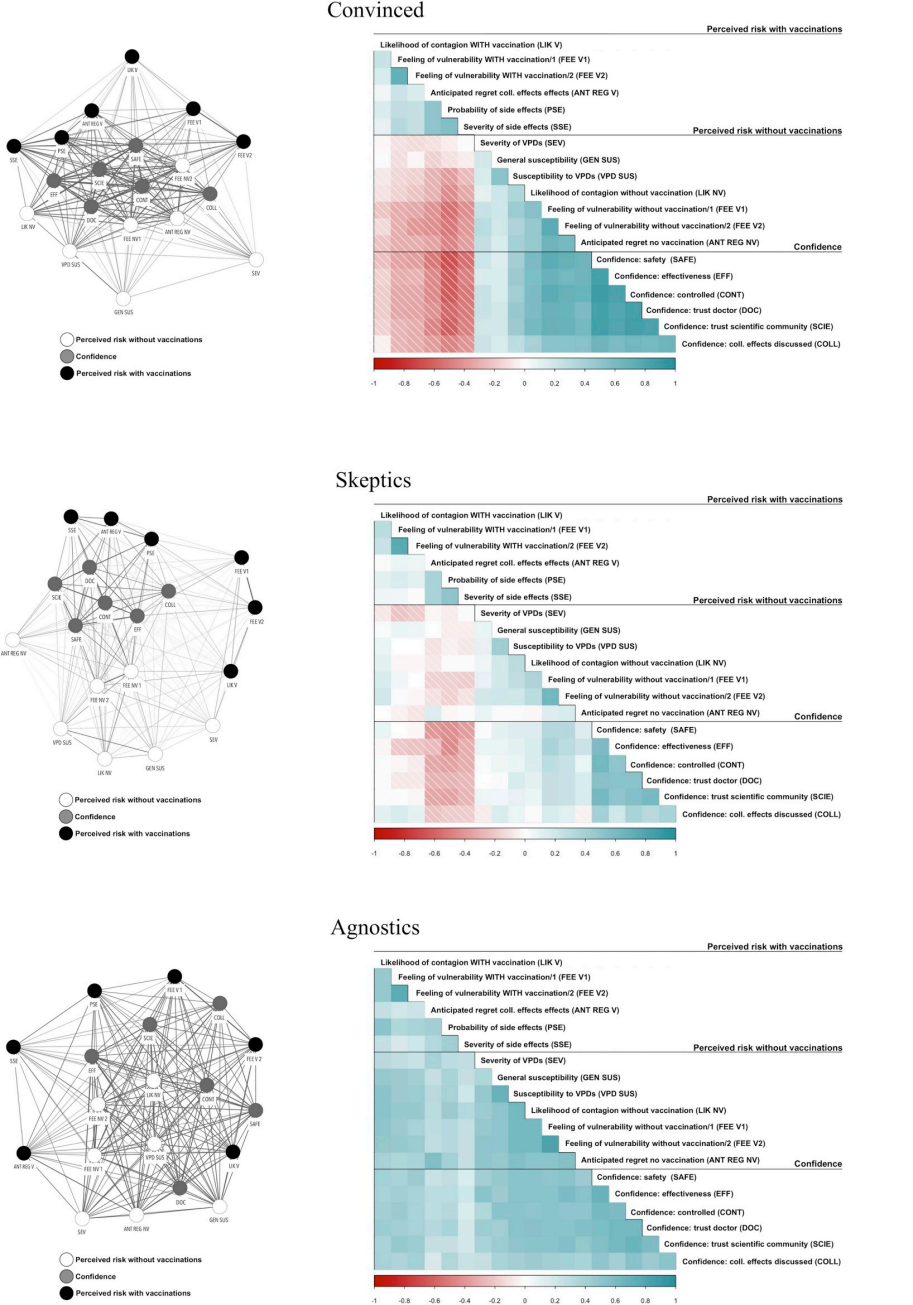
Belief networks in each group detected by RCA. In left-side network plots, each node corresponds to one variable, and each line connecting two nodes to the correlation between them, only if significant at a p ≤ 0.05. Line shades and widths are proportional to the strength of the correlation. Given the complexity of the graph, only positive correlation lines are drawn. Networks are drawn using Fruchterman-Reingold algorithm, so that distances between the nodes inversely correspond to the edge weights connecting them. On the right side, the respective correlation matrices are reported. Light blue squares represent positive correlations and red-shaded squares negative correlations. The correlation coefficient is proportional to the intensity of the respective color.

Applying RCA to the data resulted in a partition of respondents into seven groups. Four groups were removed from the analysis because they contained only one participant. The three main groups represented, respectively, 27,7 %  (n  =  279), 45,5 % (n  =  457), and 26,6 % (n  =  268) of the sample. We labeled the three groups Convinced, Skeptics, and Agnostics, respectively. The belief network for each group is represented in Fig 1.

Since RCA increases within-group covariances, correlation matrices are an informative way to represent and interpret groups’ belief networks with respect to variables involved in the analysis. Looking at correlation matrices. it is possible to deduct three different qualitative patterns of association between issue domains. The Convinced group has a decoupled position between the issue domain measuring perceived risk with vaccination on one side and risk perception without vaccination and confidence on the other. We labeled this group Convinced because they seemed to organize their representation of the vaccination issue along a line drawn in the literature between individuals in favor versus those against vaccination. This group contains, in fact, individuals who show lower perceived risk with vaccination and higher confidence and risk perception without vaccination, or vice-versa. For individuals at the extreme of this group’s continuum, we argue, vaccination can be interpreted as a protective or endangering practice, an element that reduces or increases perceived risk. For this reason, individuals in this group appeared to be firm in their belief about the positive or negative effects of vaccination. In the second group, Skeptics, respondents frame their representation of the vaccination issue as revolving around the contraposition between confidence in vaccination and the presence of side effects.

There exists, in fact, a strong negative correlation between variables assessing confidence in vaccination and three specific variables measuring the probability and severity of side effects and the anticipated regret for the side effects. Skeptics could be seen as a blurred version of the Convinced group, where individuals might apply cost/benefit reasoning, driven by the perceived level of the vaccine’s side effects. At the extremes of this group are individuals with higher confidence, higher perceived risk without vaccination, and lower perceived risk for side effects, and vice-versa. Therefore, the heterogeneity between individuals seems driven by a different perception of collateral effects and an unclear position about vaccination effectiveness in reducing the risk of VPDs. This is shown by the unclear pattern between the first three variables in the correlation matrix and the remaining ones.

The third group, Agnostics, has a significantly different pattern. The three issue domains are positively correlated with each other. At the extremes of this group’s continuum are individuals with higher confidence and higher risk perception with or without vaccination, and vice-versa. Individuals in this group appear not to recognize vaccination as a useful tool whether they perceive VPDs as dangerous and whether they trust vaccination.

Based on core predictors of vaccine acceptance, RCA revealed three different ways individuals framed their understanding of the vaccination issue, and that different cognitive schemas were indeed shared by individuals in our sample. However, the different ways perceived risk and confidence correlate in each group do not necessarily imply a difference in the levels of the variables. In other words, relational networks tell us the story about the structure of cognitive schemas, but they say little about the levels of each predictor in each group.

To understand if cognitive segmentation also identifies groups with different levels of perceived risk and confidence, we generated three standardized indexes by averaging the respondents’ opinions on the variables in each issue domain. We excluded individuals’ perceived severity of diseases, given that the severity of disease is independent of any protective measure. We additionally removed 20 cases presenting missing values on one sociodemographic variable (urban or rural area of residence) to maintain a consistent analytical sample throughout the following analysis. The indexes for confidence, risk perception without vaccination, and risk perception with vaccination show a Cronbach’s alpha scale reliability coefficient of .91, .79, and .77, and each one represented a unidimensional scale, tested through exploratory factor analysis (see S1 Fig).

**Fig 2:**
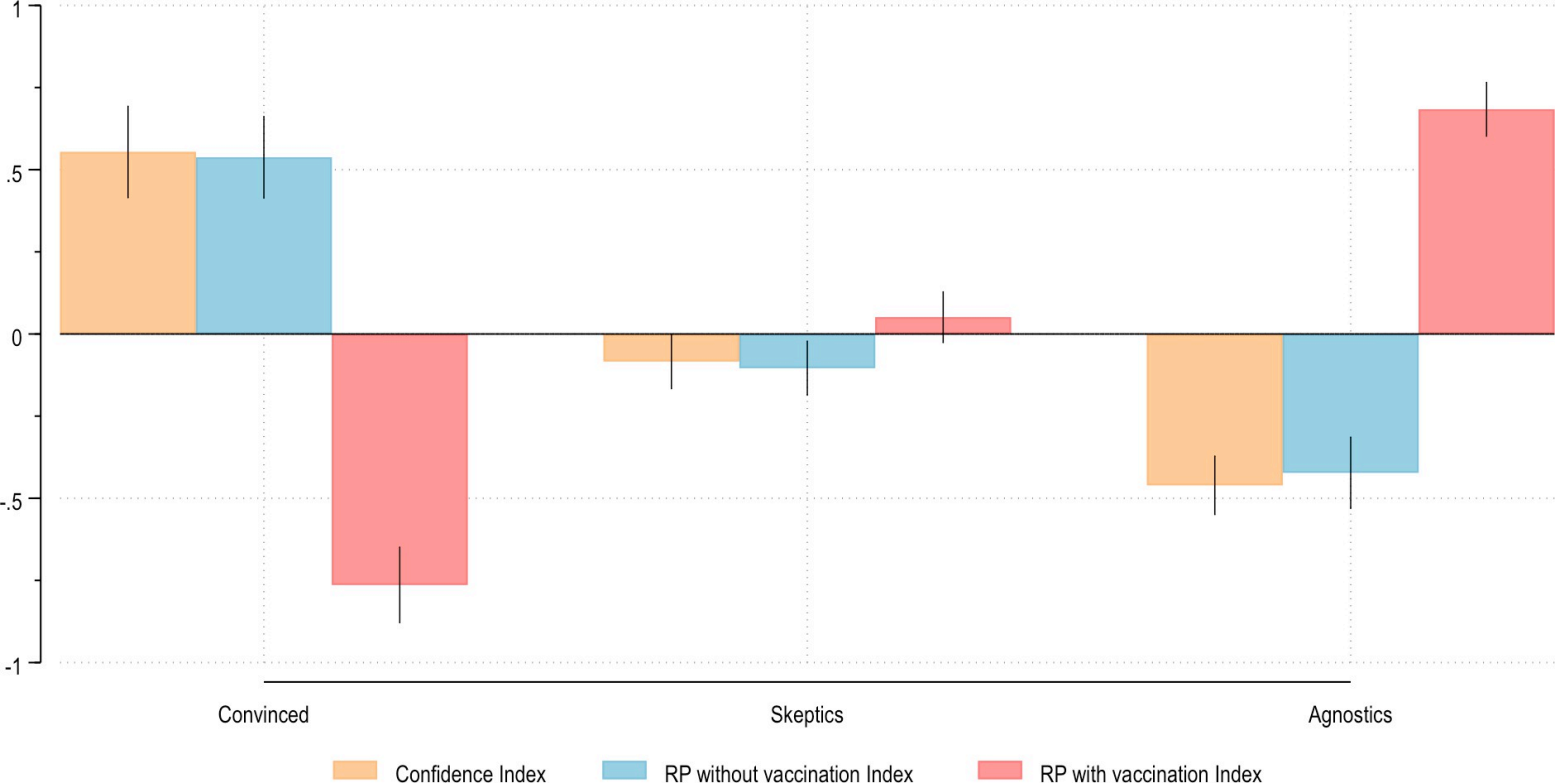
Average indexes’ levels by RCA groups. Average levels of confidence and risk perception with and without vaccination in each group detected by RCA. All standardized coefficients, 95% CIs.

Fig 2 plots the average levels of each index in each group detected by RCA.

The three groups showed significantly different levels of confidence, risk perception without vaccination, and risk perception with vaccination. The Convinced group had, on average, the highest levels of confidence in vaccination and perceived risk of VPDs without vaccination and the lowest levels of perceived risk with vaccination. This suggests that the Convinced group appeared to trust vaccines and the vaccination process, and they saw vaccination as a means to reduce perceived risk. The Skeptics showed significantly lower levels on the three indexes. Compared to the Convinced group, the Skeptics showed lower confidence in vaccination, lower perceived risk of VPDs without vaccination, and a significantly higher perceived risk with vaccination. Given the overlapping confidence intervals for perceived risk with and without vaccination, it appears that vaccination had little or no ability to reduce how this group perceived risk. Complementing RCA, a likely explanation is that for this group, fear of side effects might limit the propensity to see vaccination as an effective tool in reducing the risk of VPDs. Finally, the Agnostic group, which already appeared to answer to a very different logic, showed, on average, the lowest levels of confidence and perceived risk without vaccination. In this group, the perceived risk with vaccination was significantly higher than without, suggesting that vaccination seems not to reduce risks, but instead was a factor that contributed to defining them.

Prior research often treated vaccine hesitancy as if all individuals saw vaccination with the same eyes. RCA, on the contrary, allowed us to appreciate three systematic differences in the way individuals frame this issue by showing that individuals differ not only in “what” they think but also in “how” they think, revealing different interpretative and epistemic frameworks of the same social object. In addition, as Fig 2 points out, representational mechanisms are tied, on average, to systematically different levels in the predictors of vaccine hesitancy. This suggests that an individual’s cognitive schemas might also represent different levels of support for vaccination.

### Socio-demographic predictors of cognitive schemas

Do cognitive schemas embody principles of evaluation that demarcate different social groups? If group segmentation also represents a mean of social distinction or stratification, we should expect socio-demographic attributes to correlate with belonging to one group rather than another. On the contrary, if cognitive segmentation cuts across socio-demographic characteristics, no individual characteristic should be significantly associated with being assigned to a specific group. Using multinomial logistic regression, we analyzed the extent to which socio-demographic characteristics predicted the relative risk of belonging to each RCA group.

**Fig 3:**
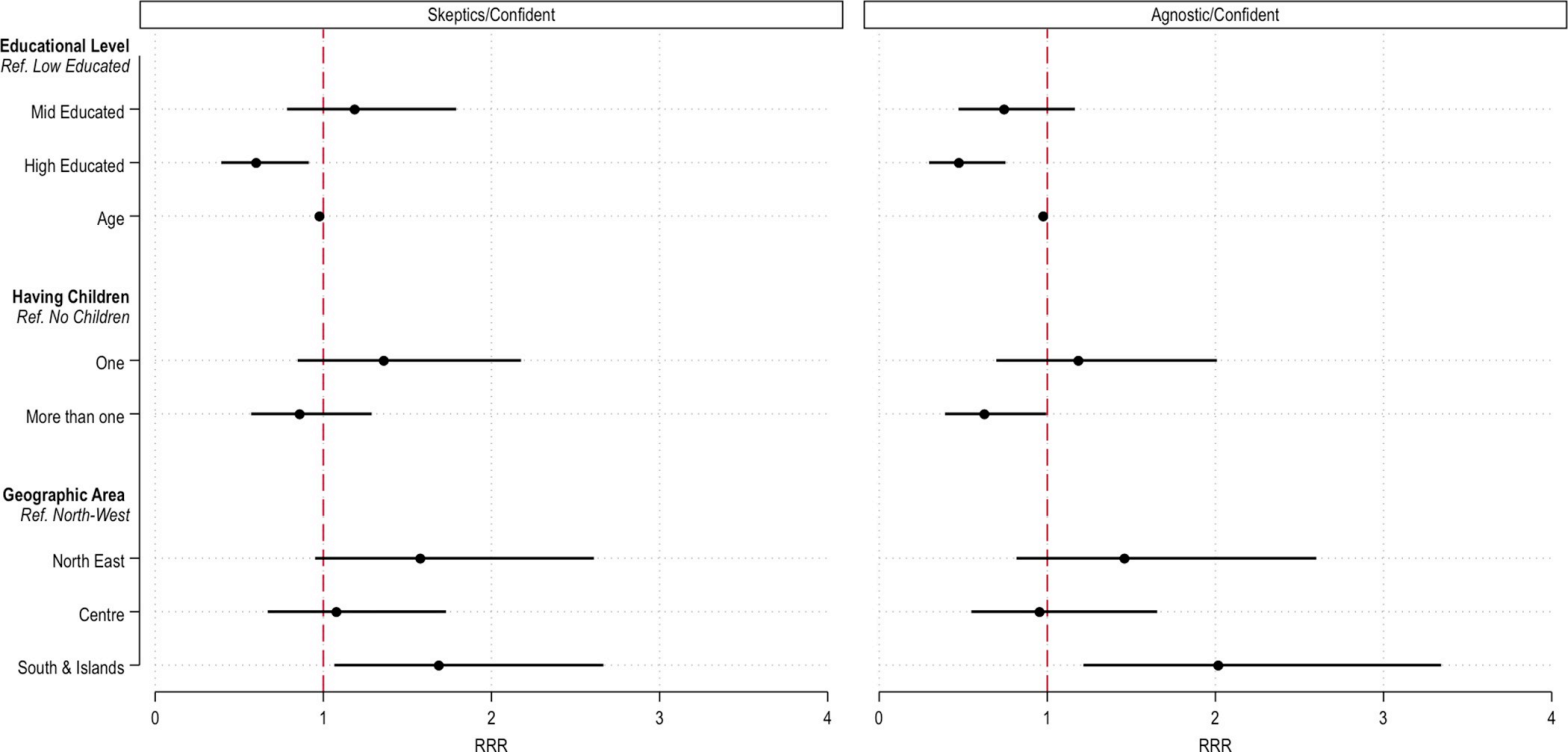
Multinomial logistic regression predicting the probability to belong to each RCA group as a function of individuals’ sociodemographic characteristics. Predictor variables include: educational level, age, gender, whether respondents have children, religious affiliation, geographic area of residence and rural or urban area of residence. Relative Risk Rations, 95% CIs, weighted coefficients.

Fig 3 plots the results of this model for statistically significant associations.

A limited number of socio-demographic characteristics were significantly associated with group belonging. Importantly, though, individuals with the highest educational level had a lower relative risk ratio of belonging to the Skeptics group or the Agnostic group, compared to the Confident group. Fig 2 showed that, on average, the three groups appeared to be in descending order of their confidence in vaccination and its ability to reduce the perceived risk of VPDs. So, it appears that a higher educational level is associated with a cognitive schema where vaccination is valued more highly. Interestingly, we also found a negative association between having more than one child and the relative risk of being in the Agnostics versus the Convinced group. This might suggest, we argue, that for Agnostics, having had one child and seeing no relevant side effects might have fostered a more favorable understanding of vaccinations. Finally, we find a geographical gradient, where individuals living in the southern regions of Italy were also more likely to be in the Skeptics and Agnostics group than the reference group.

Could these results suggest that different cognitive schemas also entail a means of social distinction or stratification? On one hand, only a few socio-demographic characteristics were significantly associated with group belonging. On the other hand, it seems important to stress that the way individuals exhibit a certain understanding of the vaccination issue is stratified by their educational level and geographical area. This might partially explain the mixed findings in the literature concerning the role of educational level. Although further research is needed here, it could be the case that the association between educational level and vaccine hesitancy might be mediated by different individual cognitive schemas and that these are distributed differently in different contexts. Overall, it is important to note that different epistemologies appeared to also provide a means of social stratification, highlighting the importance of education in favoring a worldview that supports vaccine acceptance.

### Predictors of vaccine hesitancy in each group

**Fig 4:**
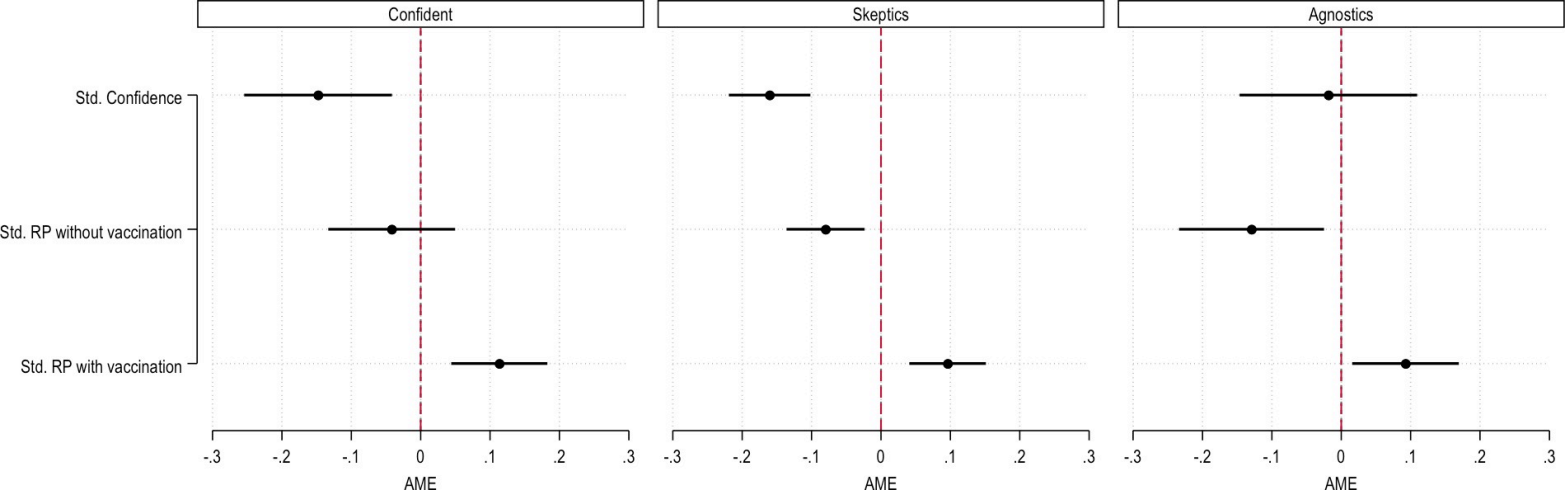
Multivariate binomial logistic regression models predicting the probability to be vaccine hesitant (0 = no hesitancy; 1 = hesitancy). Models are controlled for individuals’ sociodemographic characteristics. Average Marginal Effects. All standardized coefficients. 95% CIs, weighted coefficients.

In the third part of the analysis, we explored whether cognitive segmentation entailed a different association between predictors of vaccine hesitancy and vaccine hesitancy in each group. Fig 4 plots the average marginal effects of three binomial logistic regressions that investigated the association between the standardized indexes of issue domains and vaccine hesitancy, controlling for individuals’ socio-demographic characteristics.

Fig 4 suggests that the association between confidence, perceived risk with and without vaccination, and the likelihood of being vaccine-hesitant is articulated differently in each group. In the Confident group, higher confidence levels are associated with a lower probability of being vaccine-hesitant.

The perceived risk of VPDs in the absence of vaccination, on the contrary, was not significantly associated with a change in the probability of being vaccine-hesitant, whereas higher levels of perceived risk after vaccination are positively associated with a higher likelihood of vaccine hesitancy. The Skeptics group had a very similar scenario, but for Skeptics, higher levels of perceived risk of VPDs without vaccination were associated with a lower likelihood of being vaccine-hesitant. Finally, in the Agnostics group, confidence in vaccination, one of the main constructs analyzed in vaccine hesitancy research, appeared not to be significantly associated with the probability of being vaccine-hesitant.

This result suggests that cognitive segmentation also reveals that core predictors of vaccine acceptance are associated differently with vaccine hesitancy following individuals’ cognitive schemas. This shows the centrality of an individual’s epistemology in evaluating the dimensions of vaccine acceptance. In line with previous steps of the analysis, these findings suggest the importance of considering an individual’s cognitive characteristics in vaccine hesitancy research and in developing strategies to increase vaccine acceptance. If systematically different worldviews underpin the recognition of different elements as relevant to one’s propensity to be vaccinated, developing effective strategies to increase vaccine acceptance might require diversifying interventions based on the articulation of elements in different subgroups of the population.

## Discussion

Vaccines are safe and effective measures to prevent infectious diseases, but reaching a sufficient and stable coverage rate has proven to be more difficult than predicted. One major challenge recent research on vaccine hesitancy is facing is the recognition of the complexity and heterogeneity of the positions of individuals and the need for relevant ways to analyze them. In this paper, we argued that this heterogeneity might be investigated by exploring whether individuals show different epistemologies on the same social object by treating beliefs as relational systems.

By using fine-grained measures of predictors of vaccine hesitancy and RCA, a method tailored to identify shared cognitive structures [7], we identified three different groups of individuals, characterized by different patterns of closeness and opposition on 19 core predictors of vaccine acceptance.

Substantively, these results appear to be relevant from methodological, theoretical, and public policy perspectives. Methodologically, it tests the feasibility and usefulness of studying individuals’ opinions as relational structures on a topic such as vaccine acceptance. They revealed different patterns of association that might have gone unnoticed without such partition. In addition, the results support the idea that beyond socio-demographic and attitudinal variables, cognitive differences are a significant source of variance that should be considered when analyzing individuals’ attitudes. This also suggests the relevance of developing new strategies to disentangle individuals’ attitudes, such as RCA. Theoretically, building on the recent bridge between sociology and cognitive sciences, it highlights the importance, advantages (and complexities) of including individuals’ cognitive characteristics in the analysis of vaccine hesitancy. This is even more important in the light of the fact that we showed how cognitive segmentation identified groups with different levels of perceived risk of VPDs and confidence in vaccination (see Fig 2). Furthermore, we argue, that they are different ways to recognize vaccination as a means to reduce the risk of VPDs. Further research is needed in this liminal field, where sociology can benefit from cognitive sciences, and vice versa. From a public policy perspective, increased attention to cognitive segmentation might be another key for addressing vaccine hesitancy. Effective communication strategies to foster vaccine acceptance should consider that the ways people interact and react to public policy interventions might be conditioned not only by people’s socio-demographic profiles but also by their cultural and cognitive schemas.

In the second part of the paper, we explored whether cognitive segmentation also represented a logic of social distinction and stratification. We found that an individual’s educational level was significantly associated with being in the Convinced group, versus the Skeptics and Agnostics groups (see Fig 3). Therefore, it appears that the level of education is a powerful indicator of individuals’ worldviews, where vaccination is a trustworthy tool against VPDs. We argued in the theoretical section that there are mixed results in vaccine hesitancy research concerning the role of educational level in predicting vaccine acceptance. This is especially the case after recognizing that even highly educated individuals rely on information of questionable origin or validity [21]. This analysis, on the contrary, points toward the direction where more educated individuals are more likely to develop worldviews favorable to vaccine acceptance. This, of course, does not necessarily imply that the educational level is always a relevant predictor of vaccine hesitancy. As we pointed out, vaccine acceptance is context-specific, and given this study offers only a limited window on one European country, it is not possible to generalize results to different contexts. Nevertheless, the more nuanced result—that education is a significant predictor of a worldview where vaccination is more positively conceived—underlines that an individual’s location in our societies’ structure is still a relevant factor for vaccine acceptance. Research has often noted that vaccine acceptance is socially stratified, where subgroups in the population are systematically less likely to accept vaccination [44, 58]. Our results point toward this direction, emphasizing that to address vaccine hesitancy it is important to address deeper issues concerning our societies’ social stratification.

Finally, in the third part, we explored whether core predictors of vaccine acceptance were associated differently with vaccine hesitancy in each group. We found that higher perceived risk of VPDs with vaccination unanimously identified higher levels of vaccine skepticism. However, we did not find a significant association between the perceived risk of VPDs without vaccination in the Convinced groups and confidence in vaccination for the Agnostic group (see Fig 4). This result is important because, to increase vaccine acceptance, it is of paramount importance to identify a set of predictors on which to focus attention.

On a methodological level, this result suggests that when surveying vaccine hesitancy, conditioning perceived risk of VPDs in a scenario where vaccines have already been taken might yield hold more consistent results independently of a source of variance such as cognitive schemas. Further research should be conducted to develop valid and reliable predictors of vaccine acceptance, to make empirical results more easily comparable in and between contexts. Finally, reconstructing belief networks might help to understand the relative importance of specific predictors in different groups of individuals, a relevant toll in developing more tailored interventions.

Some limitations of this study must be considered. First, the sampling method limits the robustness of the results, and further efforts should be invested in collecting representative data to get a clearer analysis and cleaner estimates. For this reason, we strongly suggest avoiding any causal interpretation of the results and considering this exploratory study as a first step toward more ample investigations. Second, RCA is a relatively new method and its consistency should be further explored. Third, as underlined by Baldassarri and Goldberg [8], mechanically grouping individuals does not provide a straightforward interpretation of the underlying psychological mechanisms that generate this division, which remains to be tested empirically. Fourth, since data have been collected before the COVID-19 global pandemic, it is reasonable to expect that more recent information could provide a slightly different picture. Nevertheless, recent empirical contributions to the COVID-19 case underlined the importance of the dimensions highlighted in this paper [43–46, 54–56] supporting its relevance even in more recent scenarios. Finally, on the same line, the surveyed question focused on child immunization, and although the literature seems to agree on the relevance of analyzed dimensions across different recipients and vaccine types [23], results should be generalized with caution.

The contributions of this paper are threefold. First, it empirically tackled the problem of individuals’ heterogeneity on vaccine hesitancy, theoretically postulated but rarely addressed empirically. We did so by a) highlighting the importance of cognitive-based differences in the population, b) using an empirical way to address them, and c) showing how cognitive segmentation might also represent a means of social distinction. Second, it highlighted the importance of individuals’ cognitive characteristics in vaccine hesitancy research, and it advocated for a tighter connection between neighborly disciplines. Third, it contributed to the literature on measuring risk and trust in a health-related situation, recurring to an extensive set of measures that, to the best knowledge of the authors, have never been surveyed together systematically.

Further research is needed in the liminal field between social and cognitive sciences, and in developing empirical methods to analyze vaccine hesitancy further. In this paper, an exploratory analysis showed that cognitive segmentation was indeed a relevant source of variance between individuals. It also suggested that the ways people acquire, interpret, and use information might be an additional key to understanding peoples’ worldviews and fostering vaccine acceptance.

## Supporting information

S1 Table Variables description, English translation. Survey questions in Table S1 are a translation from the original Italian version. All risk perception and confidence variables included a “don’t know” option, recoded with the intermediate value.
(PDF)

S2 Table Variables description, original language. (PDF)

S3 Table Descriptive statistics for RCA variables. N = 1008. Weighted.
(PDF)

S4 Table Weighted descriptive statistics for the analytical sample and each RCA group. (PDF)

S5 Table Multinomial logistic regression model. Multinomial logistic regression predicting the relative risk ratio of belonging to “Skeptics” and “Agnostics” groups, compared to the “Confident” group as a function of individuals’ sociodemographic characteristics. Weighted coefficients. N = 984.
(PDF)

S6 Table Binomial logistic regression models. Binomial logistic regression predicting the probability to be vaccine hesitant in each RCA group. Odds ratios. Weighted coefficients.
(PDF)

S1 Fig Exploratory factor analysis for each issue domain index. Each scale displays only a single factor with eigenvalue above 1. This result is robust to different factor analysis specification.
(PDF)

S1 File Relational class analysis. (PDF)

S2 File Data. (DTA)

## References

[1] World Health Organization. Global vaccine action plan 2011–2020 [internet]. Geneva:WHO press; 2013 [cited Feb 2022]. Available from: https://www.path.org/resources/global-vaccine-action-plan-2011-2020/

[2] MacDonald N E. Vaccine hesitancy: Definition, scope and determinants. Vaccine. 2015;33(34):4161–4164. doi: 10.1016/j.vaccine.2015.04.036 25896383

[3] Larson HJ, Jarrett C, Eckersberger E, Smith DM, Paterson P. Understanding vaccine hesitancy around vaccines and vaccination from a global perspective: a systematic review of published literature, 2007–2012. Vaccine. 2014;32(19):2150–2159. doi: 10.1016/j.vaccine.2014.01.081 24598724

[4] World Health Organization. Thirteenth general programme of work, 2019–2023: promote health, keep the world safe, serve the vulnerable [internet]. Geneva:WHO press; 2019 [cited Feb 2022]. Available from: https://apps.who.int/iris/bitstream/handle/10665/324775/WHO-PRP-18.1-eng.pdf

[5] Peretti-Watel P, Seror V, Cortaredona S, Launay O, Raude J, Verger P, et al. A future vaccination campaign against COVID-19 at risk of vaccine hesitancy and politicisation. Lancet Infect Dis. 2020;20(7):769–770. doi: 10.1016/S1473-3099(20)30426-6 32445713 PMC7239623

[6] Dubé E, Ward JK, Verger P, MacDonald NE. Vaccine Hesitancy, Acceptance, and Anti-Vaccination: Trends and Future Prospects for Public Health. Annu Rev Public Health. 2021;42:175–191. doi: 10.1146/annurev-publhealth-090419-102240 33798403

[7] Goldberg A. Mapping shared understandings using relational class analysis: the case of the cultural omnivore reexamined. AJS. 2011;115(5):1397–1436.

[8] Baldassarri D, Goldberg A. Neither ideologues nor agnostics: alternative voters’ belief system in an age of partisan politics. AJS. 2014;120(1):45–95. doi: 10.1086/676042 25705780

[9] Boutyline A. Improving the measurement of shared cultural schemas with correlational class analysis: theory and method. Sociol Sci. 2017;4(15):353–393.

[10] DiMaggio P, Sotoudeh R, Goldberg A, Shepherd A. Culture out of attitudes: Relationality, population heterogeneity and attitudes toward science and religion in the US. Poetics (Amst). 2018;68:31–51.

[11] Dube E, Vivion M, MacDonald NE. Vaccine hesitancy, vaccine refusal and the anti-vaccine movement: influence, impact and implications. Expert Rev vaccines. 2015;14(1):99–117. doi: 10.1586/14760584.2015.964212 25373435

[12] Bocquier A, Ward J, Raude J, Peretti-Watel P, Verger P. Socioeconomic differences in childhood vaccination in developed countries: a systematic review of quantitative studies. Expert Rev Vaccines. 2017;16(11):1107–1118. doi: 10.1080/14760584.2017.1381020 28914112

[13] Anello P, Cestari L, Baldovin T, Simonato L, Frasca G, Caranci N, et al. Socioeconomic factors influencing childhood vaccination in two northern Italian regions. Vaccine. 2020;35(36):4673–4680. 10.1016/j.vaccine.2017.07.058 28757057

[14] Kim JJ, Andres-Beck B, Goldie S J. The value of including boys in an HPV vaccination programme: a cost-effectiveness analysis in a low-resource setting. Br J Cancer. 2017;97(9):1322–1328. 10.1038/sj.bjc.6604023 PMC2360471 17923869

[15] Pavlopoulou ID, Michail KA, Samoli E, Tsiftis G, Tsoumakas K. Immunization coverage and predictive factors for complete and age-appropriate vaccination among preschoolers in Athens, Greece: a cross-sectional study. BMC Public Health. 2013;13(1):908. doi: 10.1186/1471-2458-13-908 24083352 PMC3850659

[16] Omer SB, Salmon DA, Orenstein WA, DeHart PM, Halsey N. Vaccine refusal, mandatory immunization, and the risks of vaccine-preventable diseases. N Engl J Med. 2009;360(19):1981–1988. doi: 10.1056/NEJMsa0806477 19420367

[17] Makarovs K, Achterberg P. Contextualizing educational differences in “vaccination uptake”: A thirty nation survey. Soc Sci Med. 2017;188:1–10. doi: 10.1016/j.socscimed.2017.06.039 28692824

[18] Dubé E, Laberge C, Guay M, Bramadat P, Roy R, Bettinger J A. Vaccine hesitancy: an overview. Hum Vaccin Immunother. 2013;9(8):1763–1773. doi: 10.4161/hv.24657 23584253 PMC3906279

[19] Giambi C, Fabiani M, D’Ancona F, Ferrara L, Fiacchini D, Gallo T, et al. Parental vaccine hesitancy in Italy–results from a national survey. Vaccine. 2018;36(6):779–787. doi: 10.1016/j.vaccine.2017.12.074 29325822

[20] Carpiano RM, Bettinger JA. Vaccine coverage for kindergarteners: Factors associated with school and area variation in Vancouver, British Columbia. Vaccine Rep. 2016;6:50–55.

[21] Kirkland A. The legitimacy of vaccine critics: what is left after the autism hypothesis? J Health Polit Policy Law. 2012;37(1): 69–97. doi: 10.1215/03616878-1496020 22003097

[22] Kata A. A postmodern Pandora’s box: anti-vaccination misinformation on the Internet. Vaccine. 2010;28(7):1709–1716. doi: 10.1016/j.vaccine.2009.12.022 20045099

[23] Brewer NT, Chapman GB, Rothman AJ, Leask J, Kempe A. Increasing Vaccination: Putting Psychological Science Into Action. Psychol Sci Public Interest. 2017;18(35):149–207. 29611455 10.1177/1529100618760521

[24] Hausman BL. Anti/Vax: Reframing the vaccination controversy. Ithaca, NY: ILR Press, Cornell University Press; 2019.

[25] DiMaggio P. Culture and cognition. Annu Rev Sociol. 1997;23(1):263–287.

[26] Cerulo KA. Mining the intersection of cognitive sociology and neuroscience. Poetics (Amst). 2010;38(2):115–132.

[27] Cerulo KA, Leschziner V, Shepherd H. Rethinking Culture and Cognition. Annu Rev Sociol. 2021;47:63–85.

[28] Strandell J. Culture-Cognition Interaction: Bridging Cognitive Science and Cultural Sociology [dissertation]. Copenhagen (DK): University of Copenhagen. 2017.

[29] Fleck L. The problem of epistemology (1936). In: Cognition and Fact. Dordrecht: Springer. 1986.

[30] Berger PL, Luckmann T. The Social Construction of Reality: A Treatise in the Sociology of Knowledge. Garden City, NY: Doubleday; 1966.

[31] Merton RK. The role-set: Problems in sociological theory. Br J Sociol. 1957;8(2):106–120.

[32] Converse PE. The nature of belief systems in mass publics. In: Apter DE, editor. Ideology and Discontent. New York: Free Press. 1964.

[33] Yaqub O, Castle-Clarke S, Sevdalis N, Chataway J. Attitudes to vaccination: A critical review. Soc Sci Med. 2014;112:1–11. doi: 10.1016/j.socscimed.2014.04.018 24788111

[34] Becker MH. The health belief model and sick role behavior. Health Educ Behav. 1974;2(4):409–419.

[35] Rogers RW. A protection motivation theory of fear appeals and attitude change. J Psychol. 1975;91(1):93–114. 28136248 10.1080/00223980.1975.9915803

[36] Brewer NT, Chapman GB, Gibbons FX, Gerrard M, McCaul KD, Weinstein ND. Meta-analysis of the relationship between risk perception and health behavior: the example of vaccination. Health Psychol. 2007;26(2):136–145. doi: 10.1037/0278-6133.26.2.136 17385964

[37] Slovic P, Peters E, Finucane ML, MacGregor DG. Affect, risk, and decision making. Health Psychol. 2005;24(4S):S35. doi: 10.1037/0278-6133.24.4.S35 16045417

[38] Kahan DM. Vaccine risk perceptions and ad hoc risk communication: An empirical assessment. 2014. SSRN Electronic Journal.

[39] Weinstein ND, Kwitel A, McCaul KD, Magnan RE, Gerrard M, Gibbons FX. Risk perceptions: assessment and relationship to influenza vaccination. Health Psychol. 2007;26(2):146. doi: 10.1037/0278-6133.26.2.146 17385965

[40] Gidengil CA, Parker AM, Zikmund-Fisher BJ. Trends in risk perceptions and vaccination intentions: a longitudinal study of the first year of the H1N1 pandemic. Am J Public Health. 2012;102(4):672–679. doi: 10.2105/AJPH.2011.300407 22397349 PMC3297965

[41] Vinck P, Pham PN, Bindu KK, Bedford J, Nilles EJ. Institutional trust and misinformation in the response to the 2018–19 Ebola outbreak in North Kivu, DR Congo: a population-based survey. Lancet Infect Dis. 2019;19(5):529–536. doi: 10.1016/S1473-3099(19)30063-5 30928435

[42] Yang JZ, Chu H. Who is Afraid of the Ebola Outbreak? The Influence of Discrete Emotions on Risk Perception. J Risk Res. 2018;21(7):834–853.

[43] Attema AE, L’Haridon O, Raude J, Seror V, Peretti-Watel P, The COCONEL Group. Beliefs and Risk Perceptions About COVID-19: Evidence From Two Successive French Representative Surveys During Lockdown. Front Psychol. 2021;12:61945 doi: 10.3389/fpsyg.2021.619145 33597909 PMC7882490

[44] Dryhurst S, Schneider CR, Kerr J, Freeman AL, Recchia G, Van Der Bles AM, et al. Risk perceptions of COVID-19 around the world. J Risk Res. 2020;23(7–8):994–1006.

[45] Freeman D, Loe BS, Chadwick A, Vaccari C, Waite F, Rosebrock L, et al. COVID-19 vaccine hesitancy in the UK: the Oxford coronavirus explanations, attitudes, and narratives survey (Oceans) II. Psychol Med. 2020;1–15. 10.1017/S0033291720005188 PMC7804077 33305716

[46] Karlsson LC, Soveri A, Lewandowsky S, Karlsson L, Karlsson H, Nolvi S, et al. Fearing the disease or the vaccine: The case of COVID-19. Pers Individ Dif. 2021;172:110590. doi: 10.1016/j.paid.2020.110590 33518869 PMC7832025

[47] Larson HJ, Jarrett C, Schulz WS, Chaudhuri M, Zhou Y, Dubé E, et al. Measuring vaccine hesitancy: the development of a survey tool. Vaccine. 2015;33(34):4165–4175. doi: 10.1016/j.vaccine.2015.04.037 25896384

[48] Verger P, Dubé E. Restoring confidence in vaccines in the COVID-19 era. Expert Rev vaccines. 2020;19(11):991–993. doi: 10.1080/14760584.2020.1825945 32940574

[49] Gilkey MB, Magnus BE, Reiter PL, McRee A, Dempsey AF, Brewer NT. The Vaccination Confidence Scale: a brief measure of parents’ vaccination beliefs. Vaccine. 2014;32(47):6259–6265. doi: 10.1016/j.vaccine.2014.09.007 25258098 PMC4418546

[50] Opel DJ, Mangione R, Taylor JA, Korfiatis C, Wiese C, Catz S, et al. Development of a survey to identify vaccine-hesitant parents: the parent attitudes about childhood vaccines survey. Hum Vaccin. 2011;7(4):419–25. doi: 10.4161/hv.7.4.14120 21389777 PMC3360071

[51] Larson HJ, De Figueiredo A, Xiahong Z, Schulz WS, Verger P, Johnston IG, et al. The state of vaccine-confidence 2016: global insights through a 67-Country survey. EBioMedicine. 2016;12:295–301. doi: 10.1016/j.ebiom.2016.08.042 27658738 PMC5078590

[52] Fabry P, Gagneur A, Pasquier J-C. Determinants of A(H1N1) vaccination: cross-sectional study in a population of pregnant women in Quebec. Vaccine. 2011;29(9):1824–29. doi: 10.1016/j.vaccine.2010.12.109 21219988

[53] Rönnerstrand B. Contextual generalized trust and immunization against the 2009 A (H1N1) pandemic in the American states: A multilevel approach. SSM Popul Health. 2016;2:632–639. doi: 10.1016/j.ssmph.2016.08.004 29349177 PMC5757902

[54] Kreps S, Prasad S, Brownstein JS, Hswen Y, Garibaldi BT, Zhang B, et al. Factors associated with US adults’ likelihood of accepting COVID-19 vaccination. JAMA Netw Open 2020;3(10), e2025594–e2025594. doi: 10.1001/jamanetworkopen.2020.25594 33079199 PMC7576409

[55] Latkin CA, Dayton L, Yi G, Konstantopoulos A, Boodram B. Trust in a COVID-19 vaccine in the US: A social-ecological perspective. Soc Sci Med. 2021;270:113684. 33485008 10.1016/j.socscimed.2021.113684 PMC7834519

[56] Lazarus JV, Ratzan SC, Palayew A, Gostin LO, Larson HJ, Rabin K. A global survey of potential acceptance of a COVID-19 vaccine. Nat Med. 2021;27(2):225–228. doi: 10.1038/s41591-020-1124-9 33082575 PMC7573523

[57] Diefenbach MA, Weinstein ND, O’Reilly J. Scales for assessing perceptions of health hazard susceptibility. Health Educ Res. 1993;8(2):181–192. doi: 10.1093/her/8.2.181 10148827

[58] Robertson E, Reeve KS, Niedzwiedz CL, Moore J, Blake M, Green M, et al. Predictors of COVID-19 vaccine hesitancy in the UK household longitudinal study. Brain Behav Immun. 2021;94:41–50. doi: 10.1016/j.bbi.2021.03.008 33713824 PMC7946541

