# Supplementary material for: Shared understandings of vaccine hesitancy: How perceived risk and trust in vaccination frame individuals’ vaccine acceptance
Source: PLoS One. 2022 Oct 21;17(10):e0276519. doi: 10.1371/journal.pone.0276519 (PMC9586382; doi:10.1371/journal.pone.0276519)
Supplement: S1 Table — Survey questions in Table S1 are a translation from the original Italian version. All risk perception and confidence variables included a “don’t know” option, recoded with the intermediate value. (PDF) [file pone.0276519.s001.pdf]

**S1 Table. Variables description, English translation.** Survey questions in table S1 are a translation from the original Italian version. All risk perception and confidence variables included a "don't know" option, recoded with the intermediate value.

| Variable | Survey question | Coding/Recoding |
|---|---|---|
| *Risk perception variables* | | |
| Perceived severity (SEV) | In your opinion, how dangerous vaccine preventable diseases are? Indicate your answer in a scale from 1 to 7, where 1 corresponds to "Absolutely harmless" and 7 to "Absolutely harmful". | (1) Absolutely harmless<br>(7) Absolutely harmful<br>(88) Don't Know |
| | **Imagine you have to take care of a child, today, in Italy.** | |
| General Susceptibility (GSUS) | Could you indicate, in a scale from 1 to 7, where 1 corresponds to "Completely disagree" and 7 to "Completely agree", how much you agree or disagree with these statements?<br>Generally, the child would easily get sick | (1) Completely Disagree<br>(7) Completely Agree<br>(88) Don't Know |
| VPDs Susceptibility (VPD SUS) | Generally, the child would be exposed in this period to get a vaccine preventable disease | (1) Completely Disagree<br>(7) Completely Agree<br>(88) Don't Know |
| Likelihood of contagion without vaccines (LIK NV) | Imagine the child would have NOT received any vaccination:<br>How likely do you think it is that the child would get a vaccine preventable disease? | (1) Absolutely Unlikely<br>(7) Absolutely likely<br>(88) Don't Know |
| Feeling of vulnerability without vaccination (FEE NV1) (FEE NV2) | How much do you agree or disagree with this sentence?<br>1) Without any vaccination, I feel the child could contract a vaccine preventable disease<br>2) Without any vaccination, I feel the child would be vulnerable to vaccine preventable diseases. | (1) Completely Disagree<br>(7) Completely Agree<br>(88) Don't Know |
| Likelihood of contagion with vaccines (LIK V) | Imagine now that all mandatory and recommended vaccinations had been administer to the child:<br>How likely do you think it is that the child would get a vaccine preventable disease? | (1) Absolutely Unlikely<br>(7) Absolutely Likely<br>(88) Don't Know |
| Feeling of vulnerability with vaccination (FEE V1) (FEE V2) | How much do you agree or disagree with this sentence?<br>1) Having received all vaccinations, I feel the child could contract a vaccine preventable disease.<br>2) Having received all vaccinations, I feel the child would be vulnerable to vaccine preventable diseases | (1) Completely Disagree<br>(7) Completely Agree<br>(88) Don't Know |
| Probability of side effects (PSE) | How likely do you think it is that vaccinations might cause collateral effects? | (1) Absolutely Unlikely<br>(7) Absolutely Likely<br>(88) Don't Know |
| Severity of side effects (SSE) | How severe you think that vaccination's collateral effects could be? | (1) Absolutely Mild<br>(7) Absolutely Severe<br>(88) Don't Know |
| Anticipated regret (ANT REG NV) | Could you indicate, in a scale from 1 to 7, where 1 corresponds to "Completely disagree" and 7 to "Completely agree", how much you agree or disagree with these statements?<br>1) If I would vaccinate the child, and he/she would develop side effects, I would regret my decision to vaccinate him/her. | (1) Completely Disagree<br>(7) Completely Agree<br>(88) Don't Know |

| | | |
|---|---|---|
| Anticipated regret 2<br>(ANT REG V) | 2) If I would not vaccinate the child, and he/she would develop a vaccine preventable disease, I would regret my decision not to vaccinate him/her. | (1) Completely Disagree<br>(7) Completely Agree<br>(88) Don't Know |
| *Confidence Variables* | | |
| | Could you indicate, in a scale from 1 to 7, where 1 corresponds to "Completely disagree" and 7 to "Completely agree", how much you agree or disagree with these statements? | (1) Completely Disagree<br>(88) Don't Know<br>(7) Completely Agree |
| Safety<br>(SAFE) | Vaccines are safe | |
| Effectiveness<br>(EFF) | Vaccines are effective | |
| Controlled<br>(CONT) | Vaccines are adequately controlled before being commercialized | |
| Trust doctors<br>(DOC) | I trust my doctor's indications on vaccines | |
| Trust Scien.<br>Community<br>(SCIE) | I trust the scientific community about vaccinations | |
| Coll. Effects<br>(COLL) | Information about collateral effects are openly discussed by official authorities | |
| *Vaccine hesitancy variable* | | |
| | If you would have to decide for a child you take care of, today, in Italy, would you hesitate to administer her/him all the mandatory and recommended vaccinations indicated in the Italian vaccination plan? | (0) No hesitancy<br>(10) Max hesitancy |
| *Socio-demographic variables* | | |
| Gender | | (0) Male; (1) Female |
| Educational level | | (0) Low: up to 9 years of education<br>(1) Medium: 9-13 years of education<br>(2) High: more that 13 years of education |
| Age | | Years of age |
| Having children | | (0) Childless<br>(1) One child<br>(2) More than one child |
| Religion | Would you say you belong to a specific religion or religious denomination? | (0) No<br>(1) Yes<br>(88) I don't know |
| Urban/Rural Area | Would you say you live in: | (1) Metropolitan Area<br>(2) City/Urban centre<br>(3) Rural Area<br>(88) I don't know |
| Geographic area of residence | | (0) North-West<br>(1) North-East<br>(2) Centre<br>(3) South and Islands |
