# Supplementary material for: Shared understandings of vaccine hesitancy: How perceived risk and trust in vaccination frame individuals’ vaccine acceptance
Source: PLoS One. 2022 Oct 21;17(10):e0276519. doi: 10.1371/journal.pone.0276519 (PMC9586382; doi:10.1371/journal.pone.0276519)
Supplement: S2 Table — (PDF) [file pone.0276519.s002.pdf]

S2 Table. Variables description, original language.

Variable	Survey question	Coding/Recoding
Risk perception variables		
Perceived severity	Secondo lei, quanto solo pericolose le malattie per le quali possono essere somministrati dei vaccini? Indichi la sua risposta in base ad una scala da 1 a 7, dove 1 rappresenta "Assolutamente innocue" e 7 "Assolutamente pericolose"	(1) Assolutamente innocue<br>(7) Assolutamente pericolose<br>(88) Non so
	**Immagini di doversi prendere cura di un bambino o una bambina piccolo/a, oggi, in Italia.**	
General Susceptibility (GSUS)	Mi puo' dire, su una scala da 1 a 7, dove 1 corrisponde a " Completamente in disaccordo" e 7 a "Completamente d'accordo", quanto lei è d'accordo o in disaccordo con le seguenti affermazioni?<br>In generale, il bambino tenderebbe ad ammalarsi facilmente.	(1) Completamente in disaccordo<br>(7) Completamente d'accordo<br>(88) Non so
VPDs Susceptibility (VPD SUS)	In generale, il bambino sarebbe esposto in questo periodo a contrarre una malattia per la quale può essere somministrato un vaccino	(1) Completamente in disaccordo<br>(7) Completamente d'accordo<br>(88) Non so
Likelihood of contagion without (LIK NV)	Immagini che il bambino NON abbia ricevuto alcuna vaccinazione.<br>Quanto ritiene probabile che possa contrarre in questo periodo una malattia per la quale può essere somministrato un vaccino?	(1) Assolutamente improbabile<br>(7) Assolutamente probabile<br>(88) Non so
Feeling of vulnerability without vaccination<br>(FEE NV1)<br>(FEE NV2)	Quanto è d'accordo o in disaccordo con le seguenti affermazioni?<br>1) Senza alcuna vaccinazione, sento che il bambino potrebbe contrarre una malattia per la quale può essere somministrato un vaccino<br>2) Senza alcuna vaccinazione, sento che il bambino sarebbe vulnerabile alle malattie per le quali può essere somministrato un vaccino	(1) Completamente in disaccordo<br>(7) Completamente d'accordo<br>(88) Non so
Likelihood of contagion with<br>(LIK V)	Immagini invece che al bambino siano state somministrate tutte le vaccinazioni obbligatorie e raccomandate.<br>Quanto ritiene probabile che contragga una malattia per la quale può essere somministrato un vaccino?	(1) Assolutamente improbabile<br>(7) Assolutamente probabile<br>(88) Non so
Feeling of vulnerability with vaccination<br>(FEE V1)<br>(FEE V2)	Quanto è d'accordo o in disaccordo con le seguenti affermazioni?<br>1) Avendo ricevuto tutte le vaccinazioni, sento che il bambino potrebbe contrarre una malattia per la quale può essere somministrato un vaccino.<br>2) Avendo ricevuto tutte le vaccinazioni, sento che il bambino sarebbe vulnerabile alle malattie per le quali può essere somministrato un vaccino.	(1) Completamente in disaccordo<br>(7) Completamente d'accordo<br>(88) Non so
Probability of side effects<br>(PSE)	Quanto ritiene probabile che le vaccinazioni possano causare effetti collaterali o complicazioni?	(1) Assolutamente improbabile<br>(7) Assolutamente probabile<br>(88) Non so

Severity of side effects (SSE)	Quanto ritiene che possano essere gravi gli effetti collaterali delle vaccinazioni?	(1) Assolutamente lievi<br>(7) Assolutamente gravi<br>(88) Non so
Anticipated regret (ANT REG NV)	Mi può dire, su una scala da 1 a 7, dove 1 corrisponde a " Completamente in disaccordo" e 7 a "Completamente d'accordo", quanto è d'accordo o in disaccordo con le seguenti affermazioni?<br>1) SE NON VACCINASSI il bambino e contraesse una malattia, mi pentirei della mia decisione di non averlo vaccinato	(1) Completamente in disaccordo<br>(7) Completamente d'accordo<br>(88) Non so
Anticipated regret 2 (ANT REG V)	SE VACCINASSI il bambino e sviluppasse degli effetti collaterali mi pentirei della mia decisione di averlo vaccinato	(1) Completamente in disaccordo<br>(7) Completamente d'accordo<br>(88) Non so
Confidence Variables		
	Mi può dire, su una scala da 1 a 7, dove 1 corrisponde a " Completamente in disaccordo" e 7 a "Completamente d'accordo", quanto lei è personalmente d'accordo o in disaccordo con le seguenti affermazioni?	(1) Completamente in disaccordo<br>(7) Completamente d'accordo<br>(88) Non so
Safety (SAFE)	I vaccini sono sicuri	
Effectiveness (EFF)	I vaccini sono efficaci	
Controlled (CONT)	I vaccini sono adeguatamente controllati prima di essere immessi sul mercato	
Trust doctors (DOC)	Mi fido delle indicazioni del medico in tema di vaccinazioni	
Trust Scien. Community (SCIE)	Mi fido delle indicazioni della comunità scientifica in tema di vaccinazioni	
Coll. Effects (COLL)	Le informazioni sugli effetti collaterali dei vaccini sono discusse apertamente dalle autorità ufficiali	
Vaccine Hesitancy variable		
	Se oggi dovesse decidere per un bambino di cui si prende cura, esiterebbe nel somministrare tutte le vaccinazioni obbligatorie previste dal piano vaccinale nazionale? Me lo indichi, per favore, su una scala da 0 a 10, dove 0 rappresenta nessuna esitazione e 10 massima esitazione.	(0) Nessuna esitazione<br>(10) Massima esitazione
