# Supplementary material for: Shared understandings of vaccine hesitancy: How perceived risk and trust in vaccination frame individuals’ vaccine acceptance
Source: PLoS One. 2022 Oct 21;17(10):e0276519. doi: 10.1371/journal.pone.0276519 (PMC9586382; doi:10.1371/journal.pone.0276519)
Supplement: S3 Table — N = 1008. Weighted. (PDF) [file pone.0276519.s003.pdf]

S3 Table. Descriptive statistics for variables used in RCA analysis. N=1008. Weighted.

Variable	mean(sd)	median	min	max
LIK V	3.83 (1.76)	4	1	7
FEE V1	3.43 (1.65)	4	1	7
FEE V2	3.29 (1.63)	3	1	7
ANT REG V	4.23 (1.77)	4	1	7
PSE	3.94 (1.43)	4	1	7
SSE	4.05 (1.49)	4	1	7
SEV	5.49 (1.39)	6	1	7
GSUS	4.52 (1.48)	4	1	7
VPD SUS	4.85 (1.43)	5	1	7
LIK NV	5.21 (1.44)	5	1	7
FEE NV1	5.51 (1.36)	6	1	7
FEE NV2	5.59 (1.40)	6	1	7
ANT REG NV	5.60 (1.59)	6	1	7
SAFE	5.16 (1.45)	5	1	7
EFF	5.62 (1.28)	6	1	7
CONT	5.29 (1.47)	6	1	7
DOC	5.40 (1.36)	6	1	7
SCIE	5.29 (1.42)	6	1	7
COLL	4.61 (1.56)	5	1	7
