# Supplementary material for: Shared understandings of vaccine hesitancy: How perceived risk and trust in vaccination frame individuals’ vaccine acceptance
Source: PLoS One. 2022 Oct 21;17(10):e0276519. doi: 10.1371/journal.pone.0276519 (PMC9586382; doi:10.1371/journal.pone.0276519)
Supplement: S4 Table — (PDF) [file pone.0276519.s004.pdf]

S4 Table. Weighted descriptive statistics for the analytical sample and each RCA group.

	Analytical sample (n=984)					Confident (n=267)					Skeptics (n=450)					Agnostics (n=267)				
**Variable**	**n(%)**	**mean(SD)**	**median**	**min**	**max**	**n(%)**	**mean (SD)**	**median**	**min**	**max**	**n(%)**	**mean(SD)**	**median**	**min**	**max**	**n(%)**	**mean(SD)**	**median**	**min**	**max**
Std. R.P. without vacc. Index		-0.02 (1.00)	-0.08	-4.24	1.75		0.54 (1.04)	0.76	-3.91	1.75		-0.10 (0.89)	-0.08	-3.24	1.75		-0.42 (0.91)	-0.58	-4.24	1.75
Std. R.P. with vacc. Index		0.01 (0.99)	0.05	-2.48	2.89		-0.76 (0.96)	-0.84	-2.48	2.74		0.05 (0.84)	0.05	-2.33	2.44		0.68 (0.68)	0.50	-2.48	2.89
Std. Confidence Index		-0.02 (1.01)	0.06	-3.62	1.47		0.55 (1.16)	0.77	-3.62	1.47		-0.08 (0.90)	0.06	-3.62	1.47		-0.46 (0.74)	-0.65	-3.62	1.47
Age		43.79 (11.95)	44.00	20.00	64.00		46.45 (11.85)	48.00	21.00	64.00		42.95 (12.05)	43.00	20.00	64.00		42.69 (11.52)	43.00	20.00	64.00
Gender																				
Male	488 (49.5%)					126 (46.9%)					214 (47.4%)					149 (55.6%)				
Female	497 (50.5%)					142 (53.1%)					237 (52.6%)					119 (44.4%)				
Educational level																				
Low	352 (35.6%)					90 (33.6%)					148 (32.9%)					114 (42.6%)				
Medium	437 (44.3%)					104 (38.8%)					225 (49.8%)					108 (40.3%)				
High	196 (19.9%)					74 (27.6%)					78 (17.3%)					46 (17.1%)				
Having children																				
Zero	395 (40.1%)					95 (35.3%)					178 (39.4%)					123 (45.7%)				
One	211 (21.4%)					173 (64.7%)					273 (60.6%)					145 (54.3%)				
More than one	380(38.6%)																			
Religious																				
No	449 (45.6%)					120 (44.7%)					205 (45.3%)					126 (46.9%)				
Yes	536 (54.4%)					148 (55.3%)					246 (54.7%)					142 (53.1%)				
Geographical area																				
North-West	256 (26.0%)					86 (32.1%)					108 (24.0%)					63 (23.5%)				
North-East	184 (18.7%)					43 (16.1%)					91 (20.2%)					50 (18.4%)				
Centre	204 (20.7%)					67 (24.7%)					92 (20.4%)					47 (17.3%)				
South + Islands	342 (34.7%)					73 (27.0%)					160 (35.4%)					109 (40.8%)				
Area type																				
Metropolitan area	185 (18.7%)					59 (22.1%)					81 (18.0%)					45 (16.7%)				
City/Urban centre	524 (53.2%)					136 (50.6%)					246 (54.6%)					143 (53.2%)				
Rural area	277 (28.1%)					73 (27.3%)					124 (27.5%)					81 (30.1%)				
