# Supplementary material for: Shared understandings of vaccine hesitancy: How perceived risk and trust in vaccination frame individuals’ vaccine acceptance
Source: PLoS One. 2022 Oct 21;17(10):e0276519. doi: 10.1371/journal.pone.0276519 (PMC9586382; doi:10.1371/journal.pone.0276519)
Supplement: S5 Table — Multinomial logistic regression predicting the relative risk ratio of belonging to “Skeptics” and “Agnostics” groups, compared to the “Confident” group as a function of individuals’ sociodemographic characteristics. Weighted coefficients. N = 984. (PDF) [file pone.0276519.s005.pdf]

S5 Table. Multinomial logistic regression model. Multinomial logistic regression predicting the relative risk ratio of belonging to "Skeptics" and "Agnostics" groups, compared to the "Confident" group as a function of individuals' sociodemographic characteristics. Weighted coefficients. N= 984.

	**Skeptics/Confident b/(se)**	**Agnostics/Confident b/(se)**
Educational Level		
Ref. Cat.: Low Educated		
Mid Educated	1.184	0.741
	(0.250)	(0.171)
High Educated	0.599*	0.472**
	(0.129)	(0.112)
Gender		
Ref. Cat.: Male		
Female	0.979	0.712
	(0.170)	(0.138)
Age	0.975**	0.974**
	(0.008)	(0.009)
Having Children		
Ref. Cat.: No		
One	1.357	1.183
	(0.327)	(0.320)
More than one	0.857	0.624*
	(0.178)	(0.148)
Religious		
Ref. Cat.: No		
Yes	1.072	0.977
	(0.194)	(0.195)
Geographic Are*a*		
Ref. Cat.: North-East		
North-West	1.575	1.457
	(0.406)	(0.431)
Centre	1.076	0.952
	(0.261)	(0.268)
South and Islands	1.685*	2.015**
	(0.394)	(0.520)
Urban/Rural Area		
Ref. Cat: Metropolitan Area		
City/Urban Centre	1.129	1.102
	(0.255)	(0.288)
Rural Area	1.091	1.199
	(0.286)	(0.363)
Constant	3.965**	4.217**
	(1.832)	(2.196)

**** p<0.001, ** p<0.01, * p<0.05;* Robust SE in parentheses
