# Supplementary material for: Shared understandings of vaccine hesitancy: How perceived risk and trust in vaccination frame individuals’ vaccine acceptance
Source: PLoS One. 2022 Oct 21;17(10):e0276519. doi: 10.1371/journal.pone.0276519 (PMC9586382; doi:10.1371/journal.pone.0276519)
Supplement: S6 Table — Binomial logistic regression predicting the probability to be vaccine hesitant in each RCA group. Odds ratios. Weighted coefficients. (PDF) [file pone.0276519.s006.pdf]

S6 Table. Binomial logistic regression models. Binomial logistic regression predicting the probability to be vaccine hesitant in each RCA group. Odds ratios. Weighted coefficients.

	**Confident b/(se)**	**Skeptics b/(se)**	**Agnostics b/(se)**
Issue domains Indexes			
Std. Confidence Index	0.313*	0.406***	0.845
	(0.145)	(0.076)	(0.497)
Std. Risk without vacc. Index	0.720	0.638**	0.309*
	(0.262)	(0.106)	(0.159)
Std. Risk with vacc. Index	2.434**	1.715**	2.315*
	(0.702)	(0.286)	(0.854)
Educational Level			
Ref. Cat.: Low Educated			
Mid Educated	0.410	1.098	0.522
	(0.193)	(0.320)	(0.245)
High Educated	0.879	0.708	0.960
	(0.401)	(0.235)	(0.509)
Gender			
Ref. Cat.: Male			
Female	2.029	0.675	0.283**
	(0.867)	(0.169)	(0.124)
Age	1.032	1.022	1.000
	(0.020)	(0.012)	(0.016)
Having Children			
Ref. Cat.: No			
One	0.726	1.082	0.395
	(0.434)	(0.389)	(0.194)
More than one	0.812	0.543	0.744
	(0.359)	(0.176)	(0.398)
Religeous			
Ref. Cat.: No			
Yes	0.619	1.392	1.318
	(0.259)	(0.356)	(0.548)
Geographic Area			
Ref. Cat.: North-East			
North-West	0.163*	0.755	1.206
	(0.150)	(0.264)	(0.699)
Centre	1.282	1.155	0.995
	(0.630)	(0.404)	(0.639)
South and Islands	1.552	1.172	1.089
	(0.820)	(0.388)	(0.563)
Urban/Rural Area			
Ref. Cat: Metropolitan Area			
City/Urban Centre	1.027	0.895	1.415
	(0.513)	(0.300)	(0.737)
Rural Area	0.673	0.645	1.479
	(0.412)	(0.251)	(0.830)
Constant	0.724	0.980	4.910
	(0.815)	(0.618)	(4.705)
N	267	450	267
Pseudo R2	0.3444	0.2047	0.1935

*** $p<0.001$, ** $p<0.01$, * $p<0.05$; Robust standard errors in parentheses
