# Supplementary material for: Shared understandings of vaccine hesitancy: How perceived risk and trust in vaccination frame individuals’ vaccine acceptance
Source: PLoS One. 2022 Oct 21;17(10):e0276519. doi: 10.1371/journal.pone.0276519 (PMC9586382; doi:10.1371/journal.pone.0276519)

S1 Fig. Exploratory factor analysis for each issue domain index. Each scale displays only a single factor with eigenvalue above 1. This result is robust to different factor analysis specification.

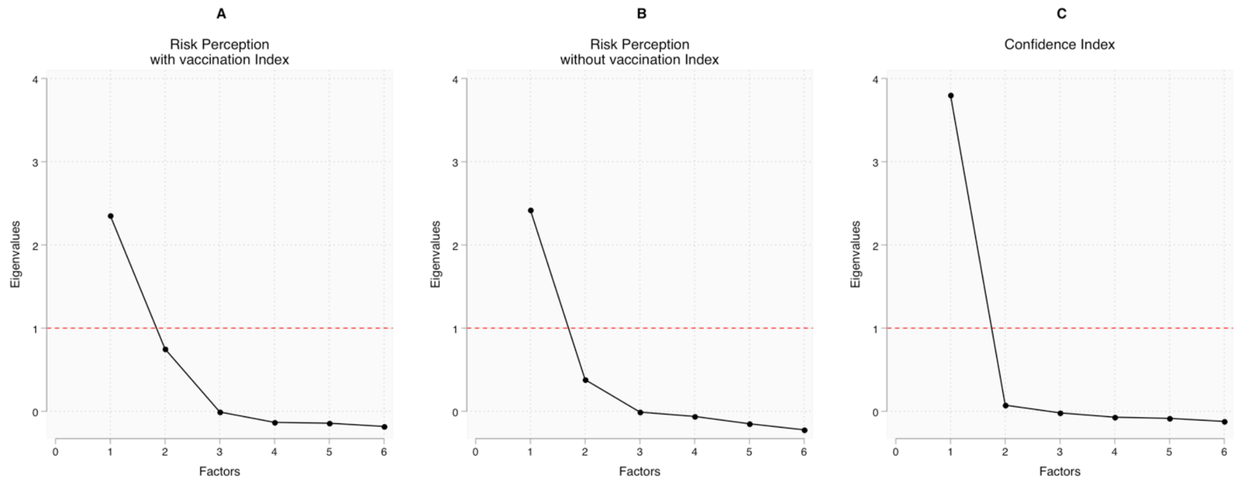

Supplement: S1 Fig — Each scale displays only a single factor with eigenvalue above 1. This result is robust to different factor analysis specification. (PDF) [file pone.0276519.s007.pdf]
