# Supplementary material for: Shared understandings of vaccine hesitancy: How perceived risk and trust in vaccination frame individuals’ vaccine acceptance
Source: PLoS One. 2022 Oct 21;17(10):e0276519. doi: 10.1371/journal.pone.0276519 (PMC9586382; doi:10.1371/journal.pone.0276519)
Supplement: S1 File — (PDF) [file pone.0276519.s008.pdf]

S1 File. Relational Class Analysis

In the first section of the paper we use Relational Class Analysis (RCA) to cluster respondents on the basis of their shared understanding of a social object. For a complete description of RCA see Goldberg (2011). In this section, we provide a brief description of RCA characteristics and functioning.

At the core of RCA are relational theories of meaning, sustaining that the meaning of symbols in a cultural system rests not in symbols themselves, but in the relationship between them (Boutyline, 2017). For example, the concept of "big" or "small" for whichever object – say, a house –, is in part defined by the relationship between the size of that object with similar ones – for example, houses in a certain neighborhood or city. Similarly, a more abstract concept such as musical taste (as in Goldberg's original example) is partly generated by the relationships between individual's appreciation or dislike for several music genres.

RCA uses this notions to 1) detect individuals "mental schemas" (Converse, 1964), 2) measure the extent to which each pair of respondents employ the same mental schema, and 3) create groups by minimizing and maximizing differences within and between groups, respectively.

To measure the extent to which two individual respondents follow the same logic or, in other words, show a similar mental schema, Goldberg (2011) uses a metric he calls "Relationality". Relationality R_{ij} between two individuals is computed as follows:

1. A row vector containing individual's responses on a series of items is taken into account.
2. Differences between respondent's values are pairwise calculated, by subtracting them from one another.
3. Each survey row i results at this point in a matrix X_i of pairwise arithmetic differences between variables in that row.

4. R_{ij} between two respondents i and j is computed as the element-wise difference between absolute values of the respective matrices X_i and X_j. Each element of the resulting matrix X_{ij} is given a sign based on whether the corresponding matrix values were in the same or opposite directions.

5. The elements of matrix X_{ij} are summed together. This results in R_{ij} value, which is rescaled ranging from -1 to 1. R_{ij} values close to the extremes suggest that vectors of responses between the two individuals i and j are similar, whereas values in between indicate that respondents present different patterns of response, showing different mental schemas (Baldassarri&Goldberg, 2014).

Relationality is then computed between each pair of individuals in the dataset, resulting in a matrix of absolute relationalities between pairwise individuals. “This matrix can be thought of as a complete non-directional weighted graph, in which each node corresponds to one observation and each edge weight is the magnitude of schematic similarity between the two observations it connects” (Goldberg, 2011:1408).

Finally, RCA uses a modularity-maximization algorithm (Newman, 2006) to partition this network into groups of respondents who have relatively high absolute relationalities, clustering together groups of respondents characterized by a similar mental schema.

References

Baldassarri, D., & Goldberg, A., 2014. Neither ideologues nor agnostics: alternative voters' belief system in an age of partisan politics. American Journal of Sociology, 120(1), 45-95.

Boutyline, A., 2017. Improving the measurement of shared cultural schemas with correlational class analysis: theory and method. Sociological Science 4(15), 353-393.

Converse, P. E., 1964. The nature of belief systems in mass publics. In Apter, D. E., (1964). Ideology and Discontent. New York: Free Press.

Goldberg, A. 2011. Mapping shared understandings using relational class analysis: the case of the cultural omnivore reexamined. American Journal of Sociology, 115(5), 1397-1436.
